# Detection by endoscopic ultrasound-guided fine-needle aspiration of retroperitoneal lymph node metastasis as the initial presentation of testicular seminoma

**DOI:** 10.1055/a-2658-0224

**Published:** 2025-08-19

**Authors:** Koichi Soga, Kazuma Sakakibara, Fuki Hayakawa, Mayumi Yamaguchi, Masaru Kuwada, Ikuhiro Kobori, Masaya Tamano

**Affiliations:** 1 26263 Department of Gastroenterology, Dokkyo Medical University Saitama Medical Center, Koshigaya, Japan

Retroperitoneal lymphadenopathy often causes suspicion of lymphoma or gastrointestinal malignancy, especially when primary lesions are not apparent. However, testicular seminoma may initially present as retroperitoneal lymph node metastasis 1 2 . We report a case of retroperitoneal seminoma in a patient without an overt testicular mass that required endoscopic ultrasound (EUS)-guided fine-needle aspiration (FNA; EUS-FNA) for an accurate diagnosis.

A 53-year-old man was referred to our hospital because of retroperitoneal lymphadenopathy. Neither upper nor lower gastrointestinal endoscopy revealed any abnormalities, and contrast-enhanced computed tomography (CT) did not reveal a clear primary tumor. However, para-aortic lymph node swelling was observed. Therefore, malignant lymphoma was initially suspected. EUS revealed a homogeneous 30-mm lymph node adjacent to the aorta, and EUS-FNA was performed using a 22-G needle. Unlike typical lymphomas, the lesion was firm during puncture ( Fig. 1 ).

**Fig. 1:**
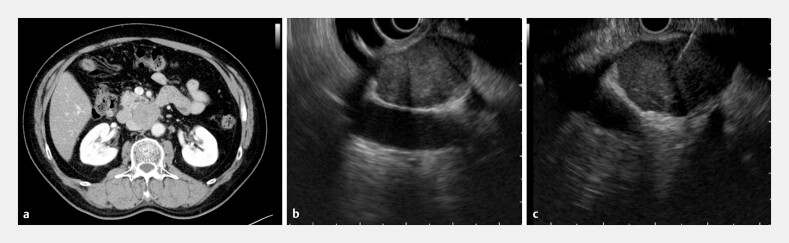
Imaging and endoscopic ultrasound (EUS)-guided fine needle aspiration (FNA; EUS-FNA) of retroperitoneal lymphadenopathy. **a** Contrast-enhanced computed tomography (CT) showed para-aortic lymph node enlargement. **b** EUS revealed a homogeneous 30-mm lymph node adjacent to the aorta. **c** EUS-FNA was performed using a 22-G needle. Unlike typical lymphomas, the lesion and node felt firm.

Histopathology demonstrated tumor cells positive for c-KIT, SALL4, and Oct-3/4 as well as those negative for AFP and CD30, consistent with seminoma. Subsequent focused CT revealed a 14-mm nodular lesion in the right testis. High inguinal orchiectomy confirmed an 11-mm pure seminoma 2 ( Fig. 2 , Video 1 ).

Retroperitoneal lymph node metastasis as the initial presentation of testicular seminoma diagnosed using endoscopic ultrasound-guided fine-needle aspiration.Video 1

**Fig. 2:**
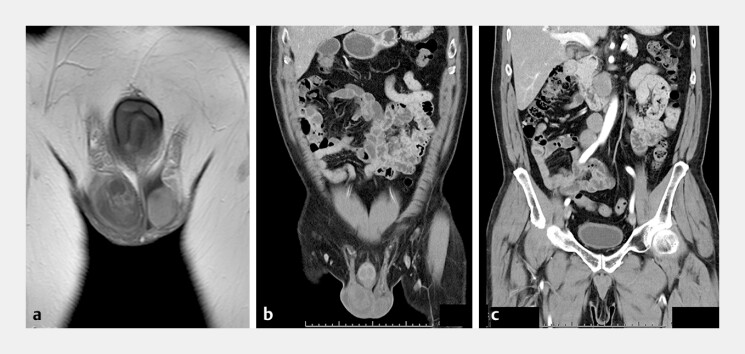
Imaging of the primary seminoma and metastatic pattern. **a** T1-weighted magnetic resonance imaging (coronal view) showed right testicular enlargement with a mosaic pattern. **b** Contrast-enhanced CT revealed a mildly hyperdense nodular lesion in the right testis. **c** CT showed lymphatic spread from the testis via the spermatic cord to the para-aortic lymph node.

This case highlights several important points. First, retroperitoneal lymphadenopathy requires a broad differential diagnosis, including lymphoma, gastrointestinal tumors, and urogenital malignancies. Second, with seminoma, metastatic lymphadenopathy can precede the identification of the primary lesion, particularly when the testicular tumor is small or regressed 1 3 . Third, lymphatic drainage from the testis follows the spermatic cord and reaches the retroperitoneal nodes near the renal hilum, thus explaining the observed distribution.

Finally, seminoma should be considered in the differential diagnosis of retroperitoneal lymphadenopathy in young and middle-aged men. A systematic diagnostic strategy that includes EUS-FNA and dedicated testicular imaging is crucial 3 . EUS-FNA plays a pivotal role in diagnosing such cases, especially when the primary lesion is inconspicuous.

Endoscopy_UCTN_Code_CCL_1AF_2AZ_3AD

## Notes

Endoscopy E-Videos https://eref.thieme.de/e-videos
*E-Videos* is an open access online section of the journal *Endoscopy* , reporting on interesting cases and new techniques in gastroenterological endoscopy. All papers include a high-quality video and are published with a Creative Commons CC-BY license. Endoscopy E-Videos qualify for HINARI discounts and waivers and eligibility is automatically checked during the submission process. We grant 100% waivers to articles whose corresponding authors are based in Group A countries and 50% waivers to those who are based in Group B countries as classified by Research4Life (see: https://www.research4life.org/access/eligibility/ ). This section has its own submission website at https://mc.manuscriptcentral.com/e-videos .
